# Transforming growth factor beta (TGF-β) induces type 1 interferon signalling in systemic sclerosis keratinocytes through the chloride intracellular channel 4 (CLIC4)

**DOI:** 10.1186/s13075-025-03632-6

**Published:** 2025-09-01

**Authors:** Christopher W. Wasson, Sophie L. Dibb, Begoña Caballero-Ruiz, Ifeoluwa E. Bamigbola, Stefano Di Donato, Eva M. Madourie-Clavane, Rebecca Wells, Vishal Kakkar, Enrico De Lorenzis, Jessica Bryon, Emma Derrett-Smith, Christopher P. Denton, Yasser El-Sherbiny, Paul J. Meakin, Rebecca L. Ross, Francesco Del Galdo

**Affiliations:** 1 Faculty of Medicine and Health, Leeds Institute of Rheumatic and Musculoskeletal Medicine, University of Leeds, Leeds, UK; 2 Biomedical Sciences in Biosciences, College of Science and Technology at Nottingham Trent University, Nottingham, UK; 3 Faculty of Medicine and Health, Leeds Institute of Cardiovascular and Metabolic Medicine, University of Leeds, Leeds, UK; 4 NIHR Leeds Musculoskeletal Biomedical Research Centre, Leeds, UK; 5 Division of Rheumatology, Catholic University of the Sacred Heart, Fondazione Policlinico Universitario A. Gemelli IRCCS, Rome, Italy; 6 Centre for Rheumatology and Connective Tissue Diseases, UCL Division of Medicine, London, UK

**Keywords:** Scleroderma, Interferon, CLIC4, STAT1

## Abstract

**Objectives:**

Systemic sclerosis (SSc) is an autoimmune disease, which is characterized by fibrosis of the skin, progressing to affect the internal organs in the most serve cases. Type 1 interferon (IFN) signalling plays a major role in SSc disease progression. The cytokine TGF-β has been extensively shown to be a major driver of fibrosis but its role in the induction of the type 1 interferon response is poorly understood.

**Methods:**

Type 1 IFN signalling was activated in keratinocytes using a range of agonists, IFN2α, Poly I:C, Poly dA:dT, LPS and TGF-β. CLIC4 activity was inhibited with the small molecule chloride channel inhibitors NPPB, IAA:94 and siRNA specific to CLIC4. Conditioned media collected from Healthy and SSc dermal fibroblasts was used to stimulate keratinocytes.

**Results:**

TGF-β stimulation induces a type 1 IFN response in keratinocytes, dependent on the chloride intracellular channel 4 (CLIC4). Inhibition of CLIC4 via small molecule inhibitors or siRNA attenuates TGF-β mediated activation of Signal Transducer and Activator of Transcription 1 (STAT1) in keratinocytes. Further analysis revealed SSc dermal fibroblasts induce a type 1 IFN response in keratinocytes in part through a TGFβR1-CLIC4 axis.

**Conclusions:**

This study shows the ability of CLIC4 to enhance TGF-β signalling is essential for aberrant type 1 interferon signalling in SSc skin.

**Supplementary Information:**

The online version contains supplementary material available at 10.1186/s13075-025-03632-6.

## Key messages

- CLIC4 is a key regulator of type-1 interferon signalling in keratinocytes
- CLIC4 enhances TGF-β signalling in keratinocytes leading to type 1 interferon signalling
- Inhibition of CLIC4 in keratinocytes blocks SSc fibroblasts from inducing type-1 interferon signalling in keratinocytes

## Introduction

Systemic Sclerosis (SSc) is an auto-immune disease which initially presents in the skin of patients and can progress to affect most internal organs. The tissue fibrosis is associated with the activation of myofibroblasts. This is regulated in part by cytokines such as IL-6 and TGF-β. In addition, a type-1 interferon signature is present in SSc skin (including VEDOSS skin) and has been associated with disease progression [1–4]. dcSSc patients with high sera levels of a panel of ISGs (CCL2, CCL8, CCL19, CXCL9, CXCL10, and CXCL11) was shown to be a predictor for high disease activity [5]. In addition, the responses of endothelial cells to IFN contributes to vasculopathy and fibrosis [6].

The role of TGF-β in SSc skin fibrosis is well characterised through its ability to activate fibroblasts, but little is known about its ability to induce a type-1 interferon response in SSc skin. There is a wealth of evidence showing TGF-β/SMAD signalling pathway can regulate IFN signalling in a range of cell types and tissues. SMAD3 can interact with Interferon Regulatory Factor 7 (IRF7) to drive IFN-β transcription [7]. Janus kinase 1 (JAK1) binds to the TGFβR1 upon activation of the receptor leading to phosphorylation of STAT1 in a SMAD independent manner [8]. STAT1 is activated by TGF-β and this activation leads to interactions between STAT1 and the TGFβR1 [9]. Recent work from our group has shown the chloride intracellular channel 4 (CLIC4) is a mediator of SSc myofibroblast activation [10, 11]. Interestingly our group and others have shown CLIC4 is an important regulator of the TGF-β signalling pathway in SSc fibroblasts and Cancer Associated Fibroblasts (CAFs) [10, 12]. Inhibition of CLIC4 was shown to attenuate TGF-β signalling in dermal fibroblasts [11]. TGF-β stimulation was also shown to trigger the nuclear translocation of CLIC4 in keratinocytes [12].

Therefore in this study, we aim to investigate the role of TGF-β and CLIC4 in regulating type-1 interferon signalling in SSc skin. We show TGF-β induces the activation of STAT1 and Interferon Stimulated Genes (ISGs) expression in keratinocytes and this is dependent of CLIC4. Further analysis revealed CLIC4 can enhance the interferon response induced by IFN agonists.

## Materials and methods

### Patient cell lines

Full thickness skin biopsies were surgically obtained from the forearms of four adult healthy controls and four adult patients with recent onset SSc, defined as a disease duration of less than 18 months from the appearance of clinically detectable skin induration. All patients satisfied the 2013 ACR/EULAR criteria for the classification of SSc as defined by LeRoy et al. [13]. All participants provided written informed consent to participate in the study. Informed consent procedures were approved by NRES-011NE to FDG. Fibroblasts and keratinocytes were isolated and established as previously described [14]. Primary cells were immortalized using human telomerase reverse transcriptase (hTERT) to produce healthy control hTERT (*N* = 4) and SSc hTERT (*N* = 4).

### Cell culture

hTERT patient fibroblasts (300,000 cells seeded per experimental condition) and the keratinocyte cell line HaCaT (600,000 cells seeded per experimental condition) (purchased from ATCC) were maintained in Dulbecco’s modified Eagle medium (DMEM) (Gibco) supplemented with 10% FBS (Sigma) and penicillin–streptomycin (Sigma). Primary SSc keratinocytes were maintained in keratinocyte growth media (Promocell). Human umbilical vein endothelial cells (HUVEC) (300,000 cells seeded per experimental condition) were maintained in ECGM2 media. Cells were treated with the chloride channel inhibitors (NPPB (25μM), IAA-94 (50μM)) or JAK1 inhibitor (Tofacitinib (2μM)) or TGF-β receptor/ALK5 inhibitor (SD-208 (1μM)) for 48h.

### Trans-well co-culture experiments

Healthy and SSc fibroblasts were seeded onto 0.4-micron pore polyethylene terephthalate (PET) transmembranes (Corning). The transmembrane was inserted in wells containing an equal number of HaCaT. After 48 h, the well was removed and the HaCaTs were harvested for protein.

### Immune agonist stimulation

Healthy dermal fibroblasts were serum starved for 24 h in DMEM containing 0.5% FBS and stimulated with Poly I:C (10 μg/ml), Poly dAdT (50 ng/ml), IFN-α2 (2 ng/ml), ODN2216 (1 μM) for 48h.

### siRNA transfections

A pool of four siRNAs specific for different regions of CLIC4 (70nM final concentration) or a negative control scrambled siRNA (Qiagen) were transfected into HaCaTs or healthy and SSc patient fibroblasts cells using Lipofectamine 2000 (Thermo Fisher). Briefly the 4 siRNAs were combined in 300μl of Opti-MEM. 1μg/ml Lipofectamine was added to the transfection mixture and incubated for 20 min. The transfection mixture was added to the cells were and incubated for 48 h prior to harvesting.

### Western blotting

Total proteins were extracted from fibroblasts in RIPA buffer and resolved by SDS-PAGE (10–15% Tris–Glycine). Proteins were transferred onto Hybond nitrocellulose membranes (Amersham biosciences) and probed with antibodies specific for α-smooth muscle actin (Abcam AB7817, 1/2000), CLIC4 (Santa Cruz sc135739, 1/2000), phosphorylated and total STAT1 (Cell signalling 9167, 9171, 1/1000), CTGF (Abcam AB209730 1/1000), phosphorylated IRF3 (Abcam AB75493, 1/1000), β-catenin (cell signalling 8480, 1/1000), phosho-SMAD3 (S423/S425) (Abcam AB52903, 1/1000), total SMAD3 (Cell signalling 9523, 1/1000), E-Cadherin (Santa Cruz sc8426, 1/1000), CXCL10 (Abcam AB9807, 1/1000), STING (Cell signalling 13,647, 1/1000) and β-Actin (Sigma A5441, 1/5000). Immunoblots were visualized with species-specific HRP conjugated secondary antibodies (Sigma) and ECL (Thermo/Pierce) on a Biorad chemiDoc imaging system. Western blot quantification was performed using Image J software.

### Quantitative real time PCR

RNA was extracted from cells using commercial RNA extraction kits (Zymo Research). RNA (1ug) was reverse transcribed using cDNA synthesis kits (Thermo). QRT-PCRs were performed using SyBr Green PCR kits on a Thermocycler with primers specific for MX1 (Forward: CGACACGAGTTCCACAAATG Reverse: AAGCCTGGCAGCTCTCTACC), CXCL10 (Forward; GGTGAGAAGAGATGTCTGAATCC Reverse; GTCCATCCTTGGAAGCACTGCA), CXCL11 (Forward; TCCCCCATGTTCAAAAGAGGAC Reverse; ATATCTGCCACTTTCACTGCTTTTAC), IFIT1 (Forward; GACTGGCAGAAGCCCAGACT Reverse; GCGGAAGGGATTTGAAAGCT), CLIC4 (Forward: CATCCGTTTTGACTTCAGTGTTG; Reverse: AGGAGTTGTATTTAGTGTGACGA) and GAPDH (Forward; ACCCACTCCTCCACCTTTGA Reverse; CTGTTGCTGTAGCCAAATTCGT). Data were analysed using the ΔΔ Ct method. GAPDH served as a housekeeping gene. The experiments were run on the Quantstudio 5 Real-Time PCR system (Thermo Fisher).

### TβRIIΔk transgenic mice experiment

All mice were housed in a clean conventional animal facility. Tissue samples were obtained from adult (age 12–18 week) sex matched littermate pairs (*n* = 3) from transgenic and non-transgenic mice genotypes by transgene specific PCR assay. The mouse strain genetic background in C57BL/6. Detailed description of the derivation and characterisation of the TβRIIΔk-fib strain [15].

### Compresstome precision Microtome

 3 mm skin biopsies were embedded in low melting point agarose. These tissue sections were sliced into 300 μm sections using the compresstome vibrating microtome (Precisionary). The sections were grown in DMEM (2% FBS) and stimulated with TGF-β (5ng/ml, Sigma Aldrich) plus NPPB for 48h. The sections were harvested for RNA.

### Immunohistochemistry

Immunohistochemistry was performed as previously described [1]. Sections were stained with CLIC4 (1/200) antibody, visualised using an HRP conjugated mouse secondary and DAB substrate. The sections were counterstained with haematoxylin. Skin sections from age matched wildtype and TβRIIΔK-fib transgenic mouse model of human SSc were also stained for CLIC4 expression in additional control experiments.

### SSc fibroblast conditioned media stimulation

Sub-confluent healthy and SSc dermal fibroblasts were grown in sera depleted DMEM for 48 h. The media was collected and centrifuged at 2000 g for 30 min. The media was added to HaCaT cells neat for 48 h or to HUVECs cells neat for 24 h.

### IRF-Lucia Luciferase reporter assay

Thp1 dual cells (Invivogen thpd-nfis) were stimulated with conditioned media from healthy and SSc dermal fibroblasts for 48 h. The media was collected from the Thp1 cells after 48 h and IRF reporter activity was measured using the QUANTI-Luc™ 4 Lucia/Gaussia, a Lucia and Gaussia luciferase detection reagent.

### Immunofluorescence

HaCaT were seeded onto coverslips. After stimulation/treatment, the cells were fixed in 4% paraformaldehyde and permeabilised with 0.1% trition- × 20 for 10 min. The cells were stained with an CLIC4 antibody and visualised with a secondary antibody conjugated to alexa-594. Nuclei were visualised by DAPI contained within the mounting media. Scale bars represent 20 μm.

### Exosome isolation and stimulation

Exosomes were isolated following the protocol previously described [16]. HaCaT were stimulated with 1% total volume of exosomes for 48 h.

### Analysis of single cell RNA sequencing dataset

The public scRNA-seq dataset GSE138699 [17], consisting of 12 systemic sclerosis (SSc) and 10 healthy control samples, was obtained from the Gene Expression Omnibus. Data processing was performed using the Seurat package v4 in R, including quality control (minGene = 200 maxGene = 5000 pctMT = 30), multi-sample integration using harmony package, normalization, and dimensional reduction (PCA and UMAP). A nearest neighbour graph was constructed using FindNeighbors, followed by clustering with FindClusters at a resolution of 0.8. Clusters were manually annotated based on top marker genes. CLIC4 expression was visualized across clusters, and statistical differences in expression levels between SSc and healthy controls in keratinocyte clusters were assessed using the Wilcoxon rank-sum test in the rstatix package.

### Statistical analysis

Data are presented as the mean ± standard error. Statistical analysis was performed using a two-tailed, unpaired Student’s *t*-test for comparisons of two groups. Multiple groups were analysed by one-way ANOVA, followed by post hoc multiple comparisons using GraphPad Prism 10.4.1.

## Results

### CLIC4 levels are elevated in SSc skin keratinocytes, fibroblasts and endothelial cells

**Fig. 1:**
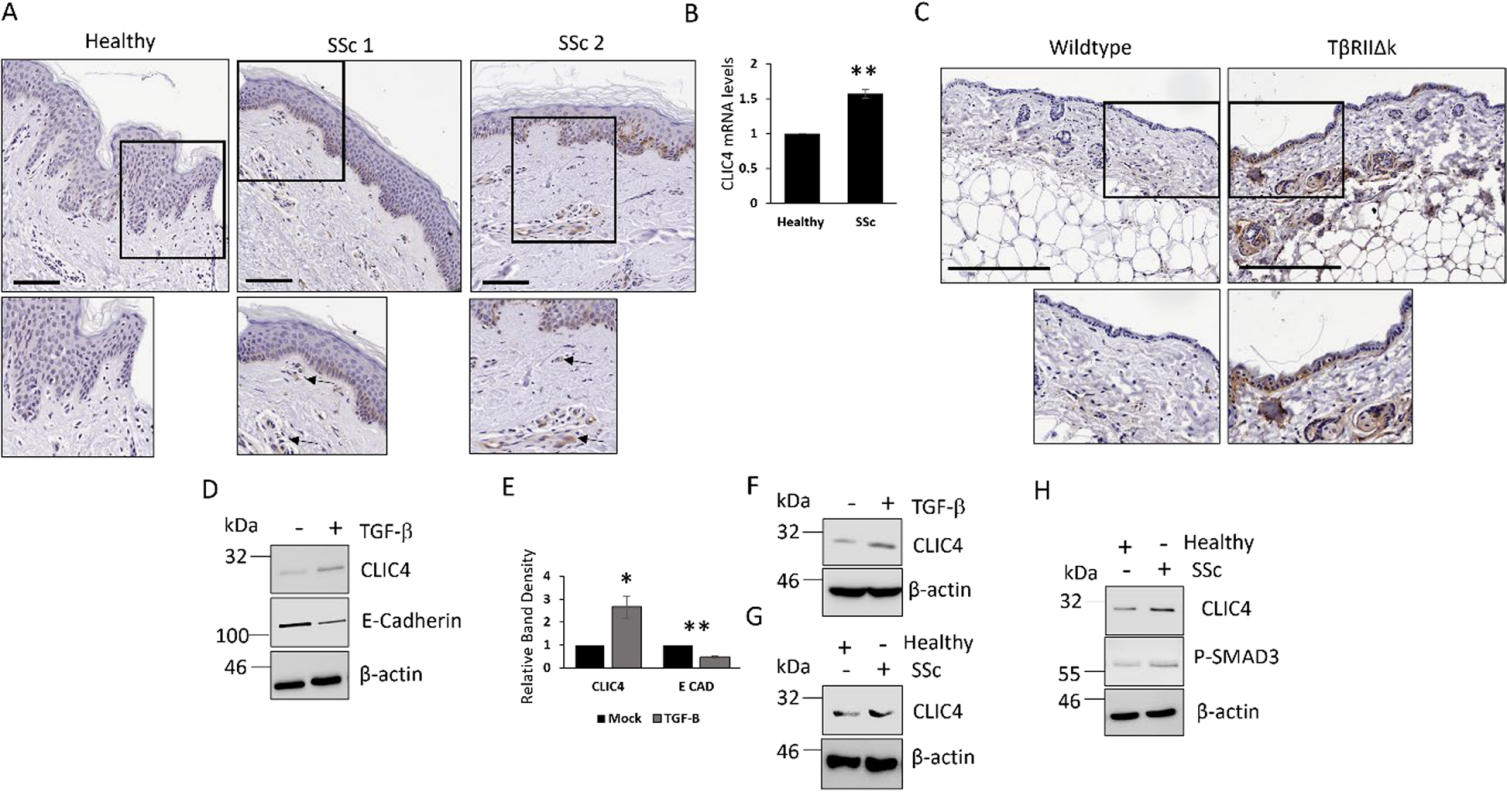
CLIC4 is elevated in SSc skin fibroblasts, keratinocytes and endothelial cells through TGF-β. **A** Skin biopsies from healthy (*N* = 4) and SSc (*N* = 4) patient were stained with an antibody specific to CLIC4. Scale bars represent 50 μm. RNA was extracted from isolated SSc skin keratinocytes. **B** CLIC4 transcript levels were analysed by qPCR. **C** Immunohistochemistry analysis for CLIC4 expression (Brown) in skin sections from age matched wildtype and TβRIIΔK-fib transgenic mice. Scale bars represent 200 μm. Protein was extracted from HaCaT (**D**-**E**) or HUVEC (**F**) cells stimulated with TGF-β for 48 h. G Protein was extracted from HaCaT that were grown in trans-wells in the presence of healthy or SSc dermal fibroblasts. H HUVECs cells were stimulated with healthy and SSc fibroblast conditioned media for 24 h. Graphs represent data from 3 independent experiments. * *p* < 0.05, ** *p* < 0.01, *** *p* < 0.001

Previously we have shown CLIC4 expression levels were increased in dermal fibroblasts isolated from SSc patient skin [10, 11] but the expression profile in SSc skin remained unknown. We analysed CLIC4 levels in SSc skin biopsies by immunohistochemistry (Fig. 1A, Supplementary Figure 1 A). We found CLIC4 levels were increased in dermal fibroblasts of the SSc skin compared to healthy control validating our previous findings [10]. Interestingly we found high levels of CLIC4 in the keratinocytes and endothelial cells in the SSc skin biopsies (Fig. 1A). CLIC4 levels were high in the lower layers of the epidermis in SSc skin (Fig. 1A). Further analysis of isolated SSc keratinocytes found *CLIC4* transcript levels were increased (1.5-fold) compared to healthy control (Fig. 1B). This was corroborated when we analysed the publicly available scRNA sequencing dataset of SSc patient skin (GSE138669) [17]. CLIC4 levels were elevated in SSc terminal keratinocytes and endothelial cells compared to healthy control (Supplementary Figure 1B).

We explored the effects of CLIC4 knockdown on pro-fibrotic signaling in SSc dermal fibroblasts with siRNA. We confirmed efficient CLIC4 knockdown in the healthy and SSc fibroblasts by western blot and qPCR (Supplementary Figure 2A-C). This knockdown of CLIC4 was able to disrupt the pro-fibrotic phenotypes in SSc fibroblasts. CLIC4 knockdown blocked pro-fibrotic marker (alpha-Smooth Muscle Actin (α-SMA) and Connective Tissue Growth Factor (CTGF) expression in SSc dermal fibroblasts (Supplementary Figure 2A-B). The disruption in CLIC4 expression also resulted in reduction of the pro-fibrotic signalling factors β-catenin (Supplementary Figure 2A, B and D), pSMAD3 (Supplementary Figure 2A-B) and GLI2 (Supplementary Figure 2A, E) but had no effect in healthy fibroblasts. These results validated previous data from our group where the CLIC4 inhibitors NPPB and IAA-94 block pro-fibrotic gene expression as well as the Wnt3a and hedgehog signalling pathways in SSc dermal fibroblasts [10, 11].

### TGF-β regulates CLIC4 levels in dermal keratinocytes and endothelial cells

CLIC4 transcription is regulated by the TGF-β mediated transcription factors SMAD2/3 in a range of cell types, including fibroblasts [10]. To determine if TGF-β drives CLIC4 expression in skin keratinocytes and endothelial cells, we analysed by immunohistochemistry skin sections from transgenic mice with fibroblast directed activation of TGF-β signalling. As previously described the TβRIIΔk mice display all the hallmarks of skin fibrosis with enhanced TGF-β signalling characterized by increased SMAD phosphorylation in fibroblasts leading paracrine activation of other cell types. This mouse strain provides a comprehensive phenocopy of human systemic sclerosis [15]. Interestingly CLIC4 levels were elevated in the fibroblasts, keratinocytes and endothelial cells of the skin in these mice (Fig. 1C). This suggests TGF-β signalling regulates CLIC4 expression in dermal keratinocytes and endothelial cells. To validate these finding we stimulated the human keratinocyte cell line HaCaT (Fig. 1D-E) and human endothelial cells HUVEC (Fig. 1F) with TGF-β and assessed CLIC4 levels. TGF-β stimulation resulted in increased CLIC4 protein levels. In addition TGF-β stimulation led to the reduction in E-Cadherin expression in the HaCaT (Fig. 1D) confirming the TGF-β was functional as it was able to induce endothelial to mesenchymal transition in the keratinocytes.

SSc fibroblasts may induce CLIC4 expression in neighboring keratinocytes and endothelial cells in SSc patient skin through paracrine signalling. To assess this hypothesis, healthy and SSc dermal fibroblasts were grown in a transwell co-culture system with HaCaT. HaCaT grown in culture with SSc dermal fibroblasts expressed higher levels of CLIC4 compared to HaCaT grown with healthy fibroblasts (Fig. 1G). Similar results were observed in HUVEC cells exposed to SSc fibroblasts (Fig. 1H). This suggests SSc fibroblasts may play a central role in regulating TGF-β signalling and in turn CLIC4 expression in neighboring cells within the skin.

### Inhibition of CLIC4 plays an important role in regulating STAT1 mediated ISG expression in keratinocytes

**Fig. 2:**
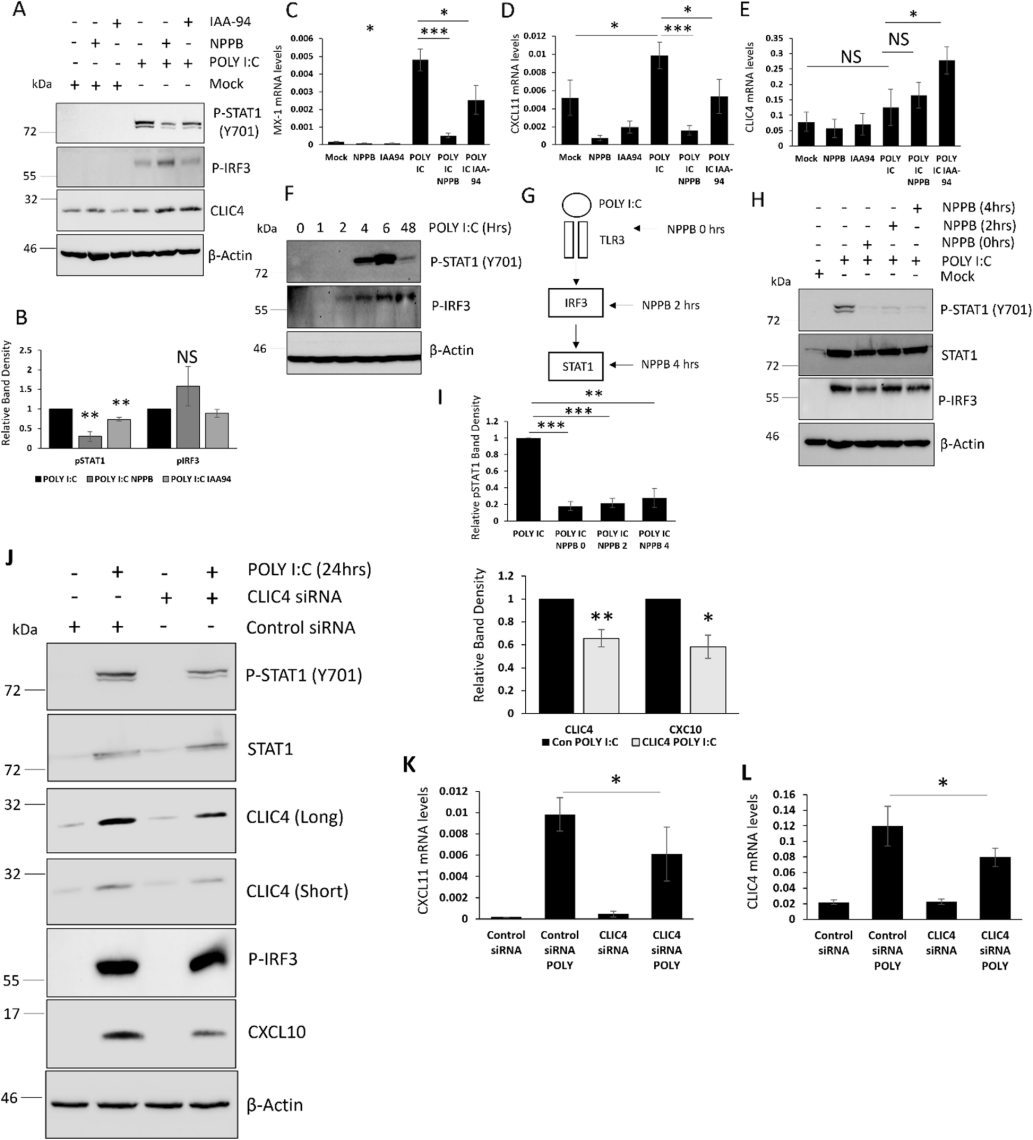
CLIC4 plays an important role in TLR3 mediated STAT1 activation in keratinocytes. **A**-**E** Protein and RNA were extracted from HaCaT stimulated with POLY I:C for 48 h. In addition, HaCaT were treated with the chloride channel inhibitors NPPB and IAA-94. **F** Protein was extracted from HaCaT stimulated with POLY I:C for 1, 2, 4, 6 and 48 h. **G**-**I** Protein was extracted from HaCaT stimulated with POLY I:C for 48 h. In addition the HaCaT cells were treated with NPPB at the same time poly I:C and 2, 4 h post POLY I:C stimulation. **J**-**L** Protein and RNA were isolated from HaCaT cells transfected with siRNA specific for CLIC4 or a scramble control siRNA for 48 h. 24 h prior to harvesting the cells were stimulated with POLY I:C. Graphs represent data from 3 independent experiments. * *p* < 0.05, ** *p* < 0.01, *** *p* < 0.001

Keratinocytes in SSc skin are a major driver of the type-1 IFN signalling and display high levels of nuclear phosphorylated STAT1 compared to healthy control skin [16]. STAT1 is a major transcription factor associated with the type-1 IFN signalling pathway. Previous studies have shown that CLIC4 is important in triggering inflammation through activation of the NLRP3 inflammasome and IL-1β in macrophages [18, 19]. Therefore, we investigated if CLIC4 could play a role in the type-1 IFN signalling observed in SSc keratinocytes. HaCaT cells were stimulated with a number of immune stimuli that trigger a type-1 IFN response in the presence and absence of the chloride channel inhibitors NPPB and IAA-94. The Toll Like Receptor 3 (TLR3) agonist POLY I:C (48 h) was able to induce STAT1 (Y701) activation in HaCaT and interestingly both chloride channel inhibitors reduced STAT1 activation (Fig. 2A-B). Next, we investigated if the inhibition of STAT1 resulted in downstream inhibition of Interferon stimulated genes (ISGs). Blocking the activity of CLIC4 resulted in reduced *MX1* and *CXCL11* expression in POLY I:C stimulated cells (Fig. 2C-D). TLR3 activation did not induce expression of CLIC4 in keratinocytes. CLIC4 protein (Fig. 2A) and transcript (Fig. 2E) levels were not altered in keratinocytes stimulated with POLY I:C for 48 h.

The Interferon regulatory transcription factor 3 (IRF3) is known to be an important component of the type-1 IFN signalling cascade and is found upstream of STAT1 in the cascade. The chloride channel inhibitors did not inhibit IRF3 phosphorylation after POLY I:C stimulation (Fig. 2A-B). This suggests CLIC4 regulates STAT1 activation downstream of IRF3. To further assess where CLIC4 intervenes in the cascade, we inhibited CLIC4 after the activation of IRF3 and STAT1. Since IRF3 and STAT1 were activated 2- and 4-h post POLY I:C stimulation respectively (Fig. 2F), we inhibited CLIC4 with NPPB, 2 and 4 h post POLY I:C stimulation (Fig. 2G). In cooperation with the data presented in Fig. 2B, NPPB blocked STAT1 activation after the activation of IRF3 (2 h). Interestingly NPPB reduced the phosphorylated STAT1 levels after its initial activation (4 h) (Fig. 2H-I). This data rules out a role for CLIC4 in regulating IFN expression or secretion and sequential IFN receptor activation and suggests inhibition of CLIC4 disrupts STAT1 phosphorylation.

### Depletion of CLIC4 plays an important role in regulating STAT1 mediated ISG expression in keratinocytes and pro-fibrotic gene expression in fibroblasts

The chloride channel inhibitors NPPB and IAA-94 target a number of other chloride channels in addition to CLIC4. Therefore to rule out off-target effects of the inhibitors we repeated the experiments above with siRNA specific to CLIC4. HaCaT cells were transfected with CLIC4 siRNA for 24 h and then the cells were stimulated with POLY I:C for a further 24 h. We observed a 45–50% knockdown in CLIC4 protein and transcript levels (Fig. 2J, L) in cells transfected with the CLIC4 siRNA. This knockdown in CLIC4 resulted in an attenuation of STAT1 activation (30% reduction) and CXCL10 expression by POLY I:C (Fig. 2J). This reduction in STAT1 activation lead to a reduction in ISG gene expression (*CXCL11* (35% reduction)) (Fig. 2K). In keeping with the inhibitor data knockdown of CLIC4 did not affect IRF3 activation in POLY I:C stimulated HaCaT (Fig. 2J). Interestingly we observed an increase in CLIC4 protein expression levels (Fig. 2J) when the HaCaT were stimulated with POLY I:C for 24 h whereas we observed no change in CLIC4 levels after 48 h stimulation (Fig. 2A). As CLIC4 protein levels change depending on the time of addition of POLY IC, we assessed CLIC4 protein levels across the POLY I:C time course (Supplementary Figure 3). Interestingly we found no change in CLIC4 expression levels in HaCaT stimulated with POLY I:C for a short period of time (1-6 h). We observed increased CLIC4 protein levels at 24 h and levels returned to unstimulated levels at 48 h. This suggests CLIC4 protein levels transiently changes during the course of the stimulation.

### CLIC4 regulates type-1 IFN signalling induced by TLR9 and cytosolic DNA sensors in keratinocytes

**Fig. 3:**
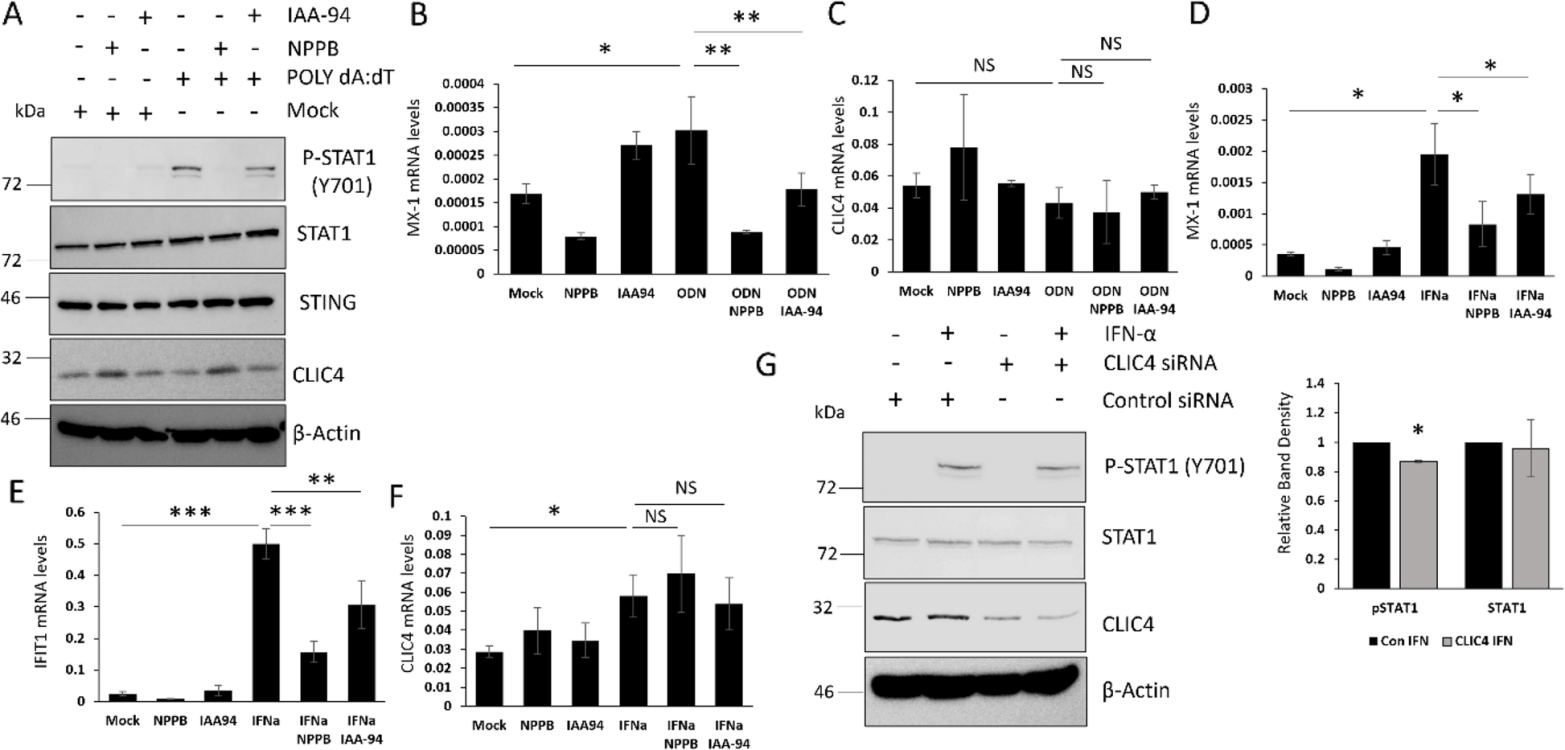
CLIC4 plays an important role for the activation of the Type 1 interferon pathway in keratinocytes. **A** Protein was extracted from HaCaT stimulated with POLY dA:dT for 48 h. In addition, HaCaT were treated with the chloride channel inhibitors NPPB and IAA-94. **B**-**C** RNA was extracted from HaCaT stimulated with ODN2216 for 48 h. In addition, HaCaT were treated with the chloride channel inhibitors NPPB and IAA-94. **D**-**F** RNA was extracted from HaCaT cells treated with the chloride channel inhibitors NPPB and IAA94 for 48 h. In addition the HaCaT were stimulated with IFN2-α for 2.5 h prior to harvesting. **G** Protein was extracted from HaCaT transfected with scramble and CLIC4 siRNA. In addition the cells were stimulated with IFN-2alpha for 2.5 h. Graphs represent data from 3 independent experiments. * *p* < 0.05, ** *p* < 0.01, *** *p* < 0.001

Next we investigated whether the inhibition of CLIC4 prevented STAT1 activation and ISG gene expression in the context of other immune stimuli or if this was specific for TLR3 (POLY I:C) signalling. We observed similar results when HaCaT cells were stimulated with the cytosolic DNA sensor agonist POLY dA:dT. Poly dA:dT stimulation resulted in activation of STAT1 in HaCaT (Fig. 3A). This was blocked in the presence of both chloride channel inhibitors. Poly dA:dT stimulation did not affect CLIC4 expression levels in HaCaT. STING is a major downstream regulator of the cytosolic DNA sensor complexes. Interestingly the chloride channel inhibitors did not affect the expression levels of STING in HaCaT cells. In collaboration with the POLY I:C data this suggests that CLIC4 specifically regulates STAT1 phosphorylation and does not affect upstream factors regardless of the stimuli. Inhibition of CLIC4 blocked TLR9 mediated ISG expression. We observed increased expression of *MX1* in ODN2216 (TLR9 agonist) treated cells, which was blocked by the chloride channel inhibitors (Fig. 3B). *CLIC4* gene expression levels were not altered in ODN2216 stimulated HaCaT (Fig. 3C). Inhibition of CLIC4 blocked the induction of *MX1* and Interferon-Induced Protein with Tetratricopeptide Repeats 1 (*IFIT1)* by IFN-2alpha in HaCaT. HaCaT were pre-treated with NPPB and IAA-94 for 45.5 h and the cells were stimulated with IFN-alpha2 for 2.5 h prior to harvesting. IFN-alpha2 increased MX1 and IFIT1 expression and this was partially blocked with both inhibitors (Fig. 3D-E). Interestingly stimulation of HaCaT with IFN-alpha for 2.5 h resulted in increased CLIC4 gene expression (Fig. 3F), further highlighting the transient nature of CLIC4 expression upon stimulation with type-1 IFN agonists. Depletion of CLIC4 from HaCaT had similar effects to the inhibitors. Depletion of CLIC4 in HaCaT attenuated the activation of STAT1 (15%) in response to IFN-2alpha (2.5-h stimulation) compared to scramble control cells stimulated with IFN-2alpha (Fig. 3G). Taken together the data here shows CLIC4 plays an important role in regulating the type-1 interferon signalling cascade regardless of the stimuli.

### Inhibition of CLIC4 blocks SSc derived STAT1 activators

**Fig. 4:**
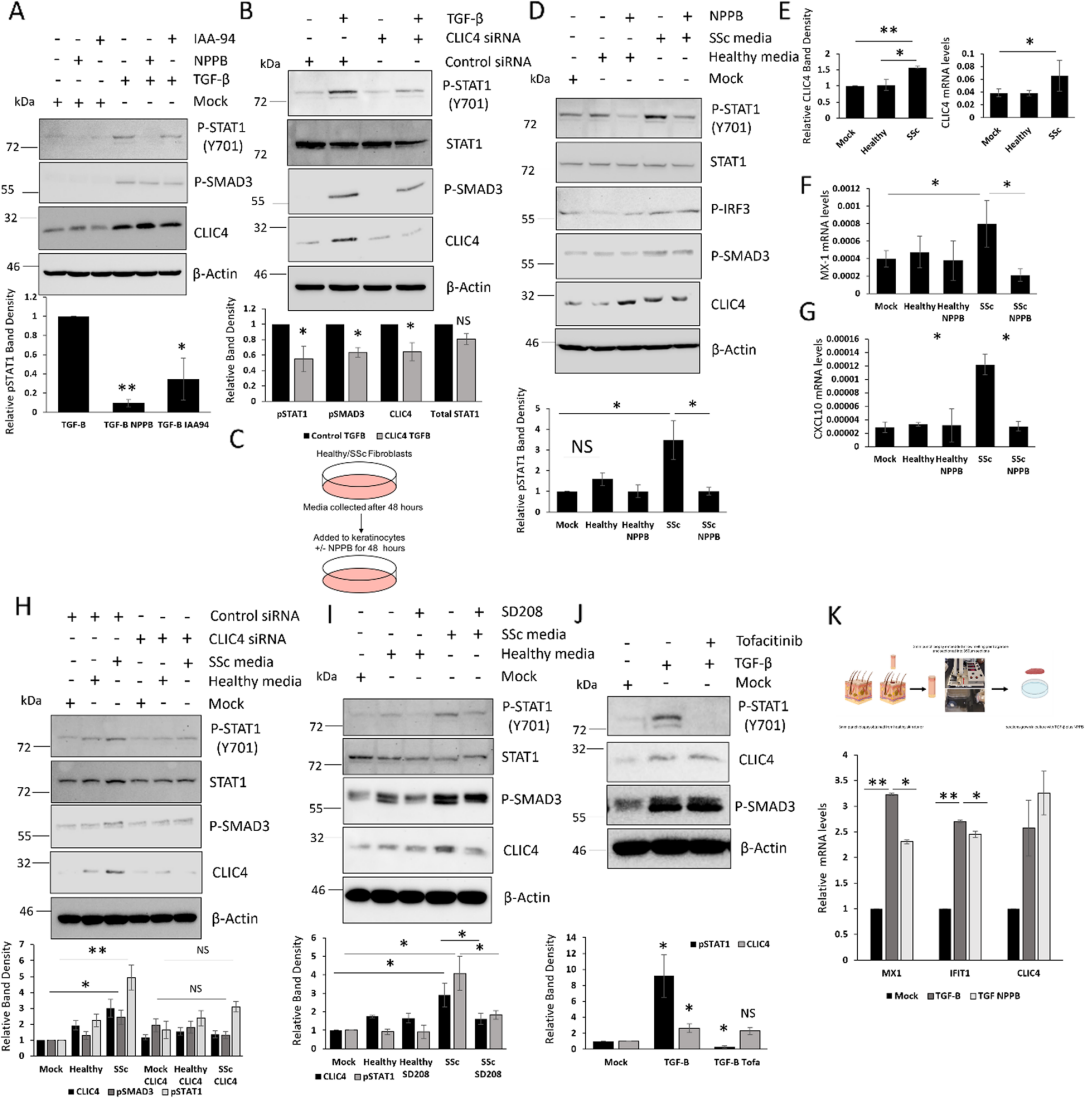
Inhibition of CLIC4 blocks SSc derived STAT1 activators. **A** Protein was extracted from HaCaT stimulated with TGF-β for 48 h. In addition, HaCaT were treated with NPPB and IAA-94. **B** Protein was extracted from HaCaT transfected with the scramble and CLIC4 siRNA. After 24 h the HaCaT were stimulated with TGF-β for a further 24 h. **C**-**G** Serum depleted conditioned media was collected from healthy and SSc patient fibroblasts after 48 h. HaCaT were stimulated with the media for 48 h in the absence or presence of NPPB. **H** HaCaT were transfected with scramble or CLIC4 siRNA for 24 h. Then stimulated with healthy or SSc fibroblast conditioned media for a further 48 h. **I** HaCaT were stimulated with healthy and SSc fibroblast media for 48 h plus/minus SD208. **J** HaCaT were stimulated with TGF-β in the absence or presence of tofacitinib for 48 h. **K** Explanted healthy skin biopsies were stimulated with TGF-β plus NPPB for 48 h. MX1, IFIT1 and CLIC4 mRNA levels were assessed. Graphs represent data from 3 independent experiments. * *p* < 0.05, ** *p* < 0.01, *** p < *0.001*

The data described above shows inhibition of CLIC4 blocked STAT1 activation by a range of immune stimuli. We next wanted to investigate the ability of the CLIC4 inhibitors to block STAT1 activation by SSc derived triggers such as TGF-β or SSc fibroblast supernatant. TGF-β is a major cytokine involved in the pathogenesis associated with SSc. TGF-β was able to induce STAT1 activation in HaCaT and this was reversed when CLIC4 is inhibited with the chloride channel inhibitors (Fig. 4A) or CLIC4 siRNA (Fig. 4B). As expected TGF-β stimulation increased CLIC4 expression and the activation of SMAD3 and this activation of SMAD3 was reduced when CLIC4 was depleted with the siRNA confirming CLIC4 role in TGF-β signalling.

Previous studies have shown SSc fibroblasts can trigger the activation of STAT1 in keratinocytes [16]. In addition, we have shown SSc fibroblast media activates IRF dependent reporter in the Thp1 monocyte cell line compared to healthy fibroblast media (Supplementary Figure 4). We therefore wanted investigated if CLIC4 was involved in the ability of SSc fibroblasts to induce type 1 interferon signalling in neighbouring keratinocytes. Conditioned media from healthy and SSc dermal fibroblasts was used to stimulate HaCaT cells for 48 h plus/minus the chloride channel inhibitor NPPB (Fig. 4C). We observed increased STAT1 phosphorylation in keratinocytes stimulated with SSc fibroblast media compared to mock and healthy fibroblast media (Fig. 4D). This was reversed when the keratinocytes were treated with the CLIC4 inhibitor NPPB. The inhibition of STAT1 lead to a 75% reduction in *MX1* and *CXCL10* transcript levels in HaCaT stimulated with SSc fibroblast media plus NPPB (Fig. 4F-G). In addition we observed an increase in phosphorylated IRF3 levels in the keratinocytes stimulated with the SSc fibroblast media but this was not altered with the addition of NPPB (Fig. 4D), further confirming that CLIC4 does not regulate IRF3 activation. We also observed that the SSc fibroblast media induced a 1.5-fold increase in CLIC4 protein (Fig. 4D) and transcript levels (Fig. 4E) in the keratinocytes. This further validates the transwell co-culture experiments in Fig. 1. In addition we observed increased nuclear staining of CLIC4 in HaCaT stimulated with SSc fibroblast media compared to healthy fibroblast media (Supplementary Figure 5) similar to that of TGF-β stimulation. This suggests that as well as increasing CLIC4 expression, SSc fibroblast media as induces the nuclear translocation of CLIC4.

Similar results were observed when conditioned media from primary healthy and SSc fibroblasts was used. Primary SSc fibroblast media induced CLIC4 expression as well as STAT1 and SMAD2/3 activation in HaCaT. The activation was reversed with NPPB (Supplementary Figure 6). Depletion of CLIC4 in HaCaT using siRNA blocked the ability of the SSc fibroblast supernatant to induce the phosphorylation STAT1 and SMAD2/3 (Fig. 4H). The media from SSc fibroblasts was able to significantly increase pSTAT1 and pSMAD3 levels (4.9-fold *p* = 0.01, 2.43-fold *p* = 0.018 respectively) compared to mock in HaCaT transfected with control siRNA but the increase in the HaCaT transfected with CLIC4 siRNA was not significant (1.88-fold *p* = 0.09, 0.68-fold *p* = 0.12 respectively).

**Fig. 5:**
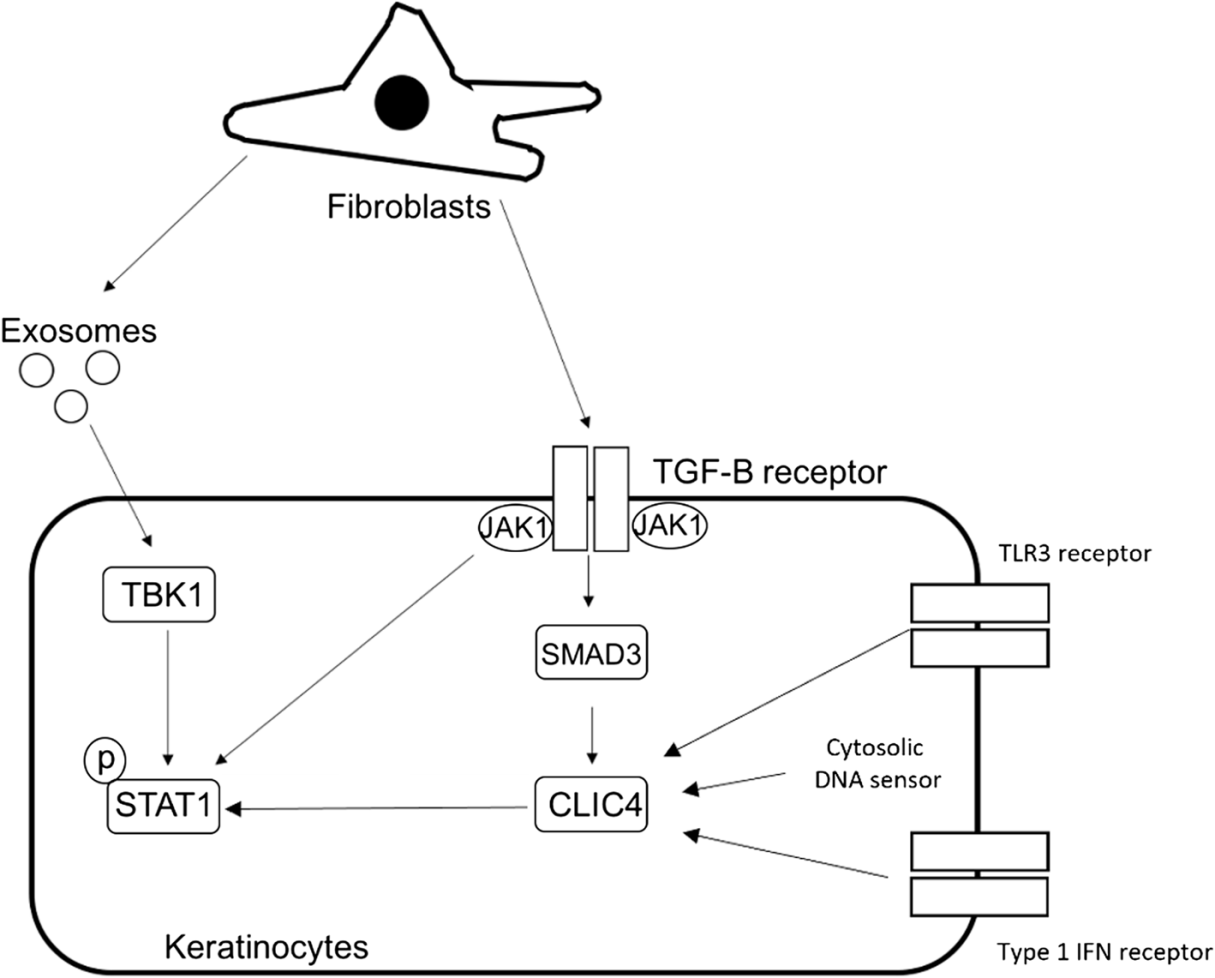
CLIC4 regulates STAT1 activation in epithelial cells through a TGF-β dependent pathway

SSc fibroblast media had the ability to induce STAT1 activation and CLIC4 expression in the keratinocytes. We therefore explored the mechanism behind the induction of CLIC4. SSc fibroblast exosomes have previously been shown to play an important role in the regulating the type 1 IFN response in the keratinocytes [16]. CLIC4 expression levels were unaffected in keratinocytes stimulated with healthy and SSc fibroblast exosomes (Supplementary Figure 7). This suggests additional factors within the SSc fibroblast supernatant drive CLIC4 expression in the keratinocytes and suggests CLIC4 and the exosomes induce type 1 interferon signaling through independent pathways.

As described above TGF-β can induce STAT1 activation in the HaCaT (Fig. 4A-B) and SSc fibroblast media is able to activate SMAD2/3 in the HaCaT (Fig. 4D, H) and the activation of both is attenuated when CLIC4 is inhibited in the HaCaT. This suggests that SSc fibroblast supernatant may induce STAT1 activation through the activation of the TGF-β receptor in HaCaT. To test this hypothesis we stimulated keratinocytes with healthy and SSc fibroblast media with the addition of the TGF-β receptor/ALK5 inhibitor SD208 (Fig. 4I). The SSc fibroblast media increased pSTAT1, CLIC4 and pSMAD3 levels in the keratinocytes and this was reversed in the presence of SD208 (Fig. 4I). Unsurprisingly this resulted in reduced *CXCL10* and *IFIT1* gene expression (Supplementary Figure 8). Further analysis revealed TGF-β receptor mediated activation of STAT1 was JAK dependent. We were able to reverse the activation of STAT1 in HaCaT stimulated with TGF-β in the presence of the specific JAK inhibitor Tofacitinib (Fig. 4J). Interestingly Tofacitinib did not block TGF-β mediated activation of SMAD2/3 or increased expression of CLIC4 (Fig. 4J). This suggests activation of the TGF-β receptor by SSc fibroblast media induces CLIC4 expression in the keratinocytes through SMAD2/3 and STAT1 is activated by SSc fibroblast media through interplay between the TGF-β receptor and JAK.

Next, we wanted to determine if CLIC4 could modulate type 1 IFN signalling in skin biopsies. Skin biopsies from healthy patients were collected and cut into 300 μm sections. These sections were grown in culture and stimulated with TGF-β with the addition of NPPB. TGF-β induced the expression of *MX1, IFIT1* and *CLIC4* in the skin biopsies (Fig. 4K). Interestingly NPPB attenuated the induction of the ISGs by TGF-β. This further confirms the importance of CLIC4 in regulating type-1 interferon signalling in skin.

## Discussion

We have shown for the first time CLIC4 is upregulated in a number of cell types within SSc patient skin and this is driven by TGF-β. CLIC4 levels were elevated in the keratinocytes and endothelial cells of TβRIIΔk mice consistent with in vivo paracrine TGF-β dependent modulation of critical cells relevant to SSc pathogenesis, as has been previously demonstrated from pulmonary epithelial and endothelial compartments in this mouse strain [20]. We identified cell to cell communication between the fibroblasts, keratinocytes and endothelial cells was important for regulating CLIC4 expression. Keratinocytes grown in a transwell co-culture system with SSc fibroblasts induced CLIC4 expression (Fig. 1C) and conditioned media from SSc fibroblasts was able to induce CLIC4 expression in keratinocytes and endothelial cells. This was dependent on the activation of the TGF-β receptor in the keratinocytes as the TGF-β receptor/ALK5 inhibitor SD208 blocked CLIC4 expression induced by the SSc fibroblast conditioned media (Fig. 4I). Although we have not explored the role of CLIC4 in endothelial cells in the context of SSc in this study, it is interesting that aberrant expression of CLIC4 has previously been implicated in Pulmonary arterial hypertension (PAH) [21] which hints at a role for CLIC4 in SSc endothelial cell dysfunction [22]. One caveat of this study was that the immortalised keratinocyte cell line HaCaT were used. Future work will explore the effects on primary keratinocytes.

We believe CLIC4 modulates the type-1 IFN pathway by enhancing STAT1 phosphorylation. The CLIC4 inhibitors blocked the phosphorylation of STAT1 but do not affect upstream proteins such as IRF3. Furthermore, time course experiments (Fig. 2G-H) showed the addition of the inhibitors after the activation of STAT1 (4 h) by POLY I:C led to a blockage of said activation of STAT1. This is further evidence that CLIC4 regulates the type-1 IFN signalling through STAT1.

The ability of the CLIC4 inhibitors to block SSc fibroblast mediated STAT1 activation in the keratinocytes is intriguing. SSc fibroblast conditioned media and exosomes have previously been shown to induce STAT1 activation through the TBK1 [16]. Data presented in this study shows CLIC4 is not an intermediator of this pathway as CLIC4 expression levels were unaffected in keratinocytes stimulated by SSc fibroblast exosomes. Our hypothesis is the SSc fibroblast exosomes trigger the activation of STAT1 in keratinocytes and CLIC4 amplifies or maintains this activation leading to the dysregulated type-1 IFN signalling in SSc skin (Fig. 5).

Furthermore, we have shown for the first time that SSc fibroblasts induce the type-1 IFN response in keratinocytes in part through the activation of the TGF-β signalling cascade and this activation is mediated through JAK. This is interesting as previous studies have shown JAK1 can bind to the TGF-β receptor and induce STAT activation in a SMAD independent manner [8]. This corroborates data presented in this study where TGF-β mediated activation of STAT1 is disrupted by the JAK inhibitor Tofacitinib but the inhibitor does not affect SMAD2/3 activation or CLIC4 expression (Fig. 4J). At the same time the SSc fibroblast media induces CLIC4 expression through TGF-β/SMAD dependent signalling. CLIC4 could then enhance STAT1 activation through the inhibition of the protein phosphatase 1a (PPM1a). Nuclear CLIC4 binds to PPM1a and prevents de-phosphorylation of p38 in fibroblasts [23] thus enhancing TGF-β signalling in both cell types. Interestingly PPM1a can also target and inhibit STAT1 in monocytes [24]. Inhibition of PPM1a in monocytes leads to increased STAT1 activation. Therefore, it is possible that the CLIC4 inhibits PPM1a in keratinocytes by binding to PPM1a and preventing it interacting with its substrates which would lead to elevated STAT1 activation.

SSc keratinocytes have been shown to induce activation of neighboring fibroblasts [25] and this was shown to be through NF-κB and independent of TGF-β. Therefore it would be interesting to investigate the role of CLIC4 in this aspect of keratinocyte/fibroblast crosstalk in SSc skin. CLIC4 has previously been shown not to activate NF-κB [19] therefore it is likely CLIC4 role is limited.

In conclusion, the elevated type-1 IFN signalling found in the epidermis of SSc skin may in part be due to increased CLIC4 expression in the epidermis which could lead to increased STAT1 activation and expression of ISGs. Targeting CLIC4 in SSc may prevent the inflammation associated with SSc and the fibrotic element of the disease [10, 11]. Therefore, designing specific inhibitors that target CLIC4 ion channel function is a potential future therapeutic avenue to explore.

## Supplementary Information

Supplementary Material 1. Supplementary Fig. 1: CLIC4 expression is upregulated in SSc skin keratinocytes. (A) Skin biopsies from healthy and SSc patient forearms were stained with an antibody specific to CLIC4 and visualized with an HRP conjugated secondary antibody. Scale bars represent 50 μM. (B) Analysis of CLIC4 expression levels in public SSc skin single cell RNA-sequencing dataset (GSE138669).

Supplementary Material 2. Supplementary Fig. 2: Knockdown of CLIC4 disrupts the pro-fibrotic phenotype in SSc dermal fibroblasts. Protein and RNA were extracted from healthy and SSc dermal fibroblasts transfected with siRNA specific for CLIC4. (A) pSMAD3, total SMAD3, β-catenin, GLI2, CTGF, α-SMA and CLIC4 protein levels were assessed by western blot. β-actin was used as a loading control. (B) Graph represent densitometry analysis for the mean and standard error for three independent experiments. CLIC4 (C), β-catenin (D) and GLI2 (E) transcript levels were assessed by qPCR. * *p* < 0.05, ** *p* < 0.01, *** *p* < 0.001.

Supplementary Material 3. Supplementary Fig. 3: POLY I:C induces CLIC4 expression in a time dependent manner. (A) HaCaT cells were stimulated with POLY I:C for between 1-48 h. Protein was extracted from the cells and pSTAT1, CLIC4 and pIRF3 protein levels were analysed by western blot. β-actin was used as a loading control. Graphs represent densitometry analysis for the mean and standard error for three independent experiments.

Supplementary Material 4. Supplementary Fig. 4: SSc fibroblast conditioned media induces IRF promoter activation in reporter cells. Thp1 dual luciferase reporter cells were grown in transwell co-culture with healthy and SSc dermal fibroblasts. Supernatant from the Thp1 cells was collected and IRF reporter activity was measured by QUANTI-Luc™ 4 Lucia/Gaussia. * *p* < 0.05, ** *p* < 0.01, *** *p* < 0.001.

Supplementary Material 5. Supplementary Fig. 5: SSc fibroblast conditioned media induces CLIC4 nuclear expression in HaCaTs. HaCaT were stimulated with conditioned media from healthy and SSc dermal fibroblasts for 48 h. In addition HaCaTs were stimulated with TGF-β for 48 h. The cells were stained with an antibody specific for CLIC4 and visualized with an alexa-594 antibody. Nuclei were visualized with DAPI.

Supplementary Material 6. Supplementary Fig. 6: Inhibition of CLIC4 blocks SSc derived STAT1 activators. Serum depleted conditioned media was collected from primary healthy and SSc patient fibroblasts after 48 h. HaCaT were stimulated with the media for 48 h in the absence or presence of NPPB. pSTAT1, STAT1 pIRF3, pSMAD3 and CLIC4 protein levels were assessed by western blot. β-actin was used as a loading control.

Supplementary Material 7. Supplementary Fig. 7: SSc fibroblast exosomes do not stimulate CLIC4 expression in keratinocytes. Exosomes isolated from healthy and SSc patient fibroblasts were used to stimulate HaCaT for 48 h. (A) RNA was isolated from stimulated HaCaT and CLIC4 transcript levels were assessed. (B) Protein was isolated from stimulated HaCaT and CLIC4 protein levels were assessed by western blot. Graphs represent the mean and standard error for densitometry analysis.

Supplementary Material 8. Supplementary Fig. 8: Inhibition of the TGF-β signaling attenuates SSc fibroblast mediated activation of type 1 interferon in keratinocytes. Serum depleted conditioned media was collected from primary healthy and SSc patient fibroblasts after 48 h. HaCaTs were stimulated with the media for 48 h in the absence or presence of SD208. CXCL10 (A) and IFIT1 (B) transcript levels were assessed. * *p* < 0.05, ** *p* < 0.01, *** *p* < 0.001.

Supplementary Material 9.

## Data Availability

No datasets were generated or analysed during the current study.
