# Supplementary figures and images for: Transforming growth factor beta (TGF-β) induces type 1 interferon signalling in systemic sclerosis keratinocytes through the chloride intracellular channel 4 (CLIC4)
Source: Arthritis Res Ther. 2025 Sep 1;27:173. doi: 10.1186/s13075-025-03632-6 (PMC12400655; doi:10.1186/s13075-025-03632-6)

A

Healthy

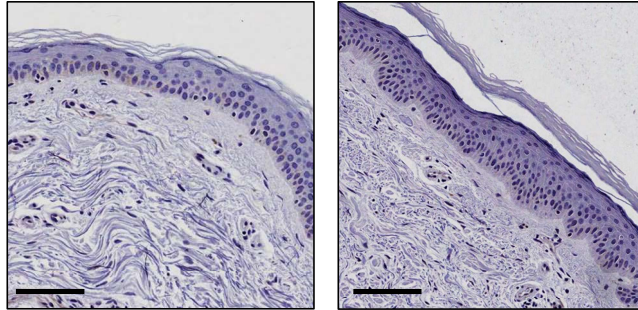

SSc

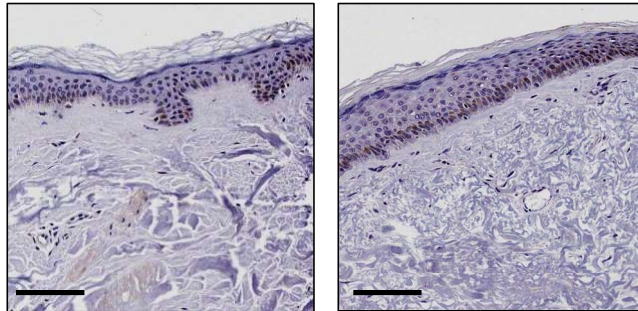

SSc

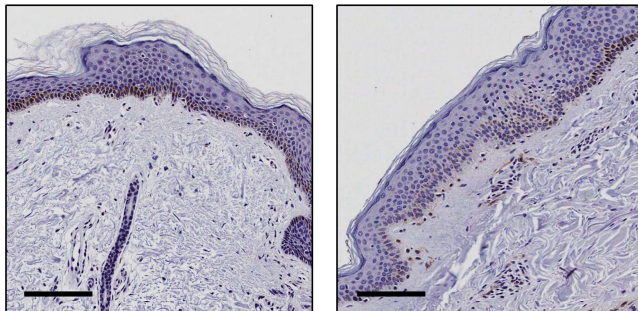

B

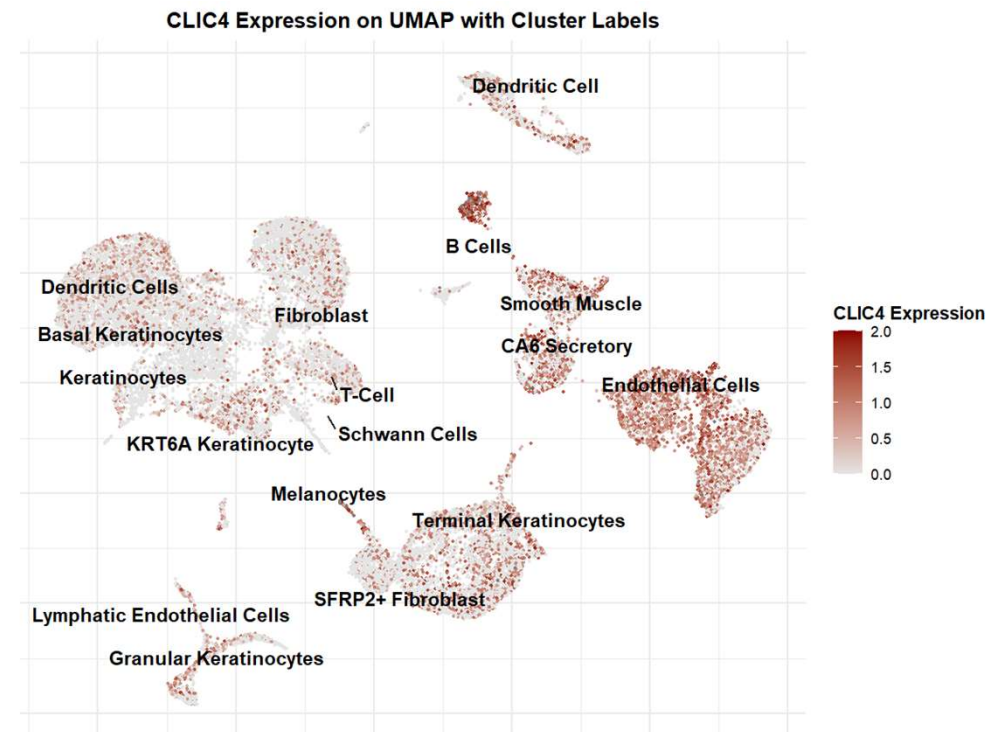

Supplementary Figure 1: CLIC4 is upregulated in SSc skin keratinocytes

Supplement: Supplementary file 1 — Supplementary Material 1. Supplementary Fig. 1: CLIC4 expression is upregulated in SSc skin keratinocytes. (A) Skin biopsies from healthy and SSc patient forearms were stained with an antibody specific to CLIC4 and visualized with an HRP conjugated secondary antibody. Scale bars represent 50 μM. (B) Analysis of CLIC4 expression levels in public SSc skin single cell RNA-sequencing dataset (GSE138669). [file 13075_2025_3632_MOESM1_ESM.pdf]

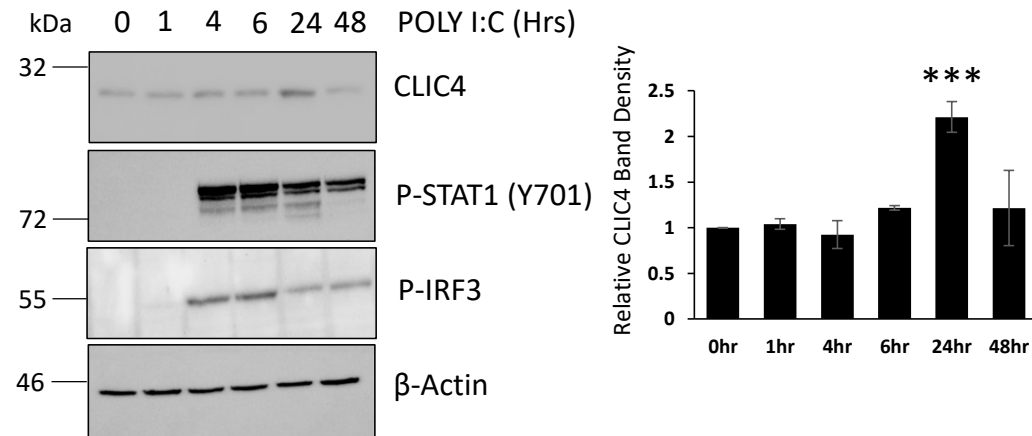

Supplementary Figure 3: POLY I:C induces CLIC4 expression in a time dependent manner

Supplement: Supplementary file 3 — Supplementary Material 3. Supplementary Fig. 3: POLY I:C induces CLIC4 expression in a time dependent manner. (A) HaCaT cells were stimulated with POLY I:C for between 1-48 h. Protein was extracted from the cells and pSTAT1, CLIC4 and pIRF3 protein levels were analysed by western blot. β-actin was used as a loading control. Graphs represent densitometry analysis for the mean and standard error for three independent experiments. [file 13075_2025_3632_MOESM3_ESM.pdf]

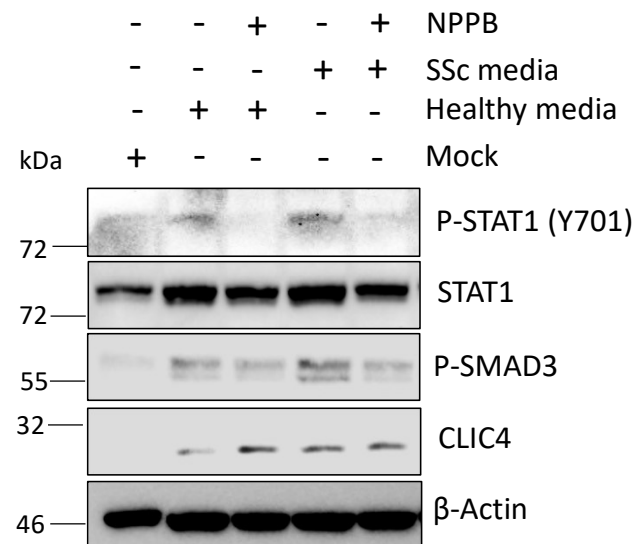

Supplementary Figure 6: Inhibition of CLIC4 blocks SSc derived STAT1 activators

Supplement: Supplementary file 6 — Supplementary Material 6. Supplementary Fig. 6: Inhibition of CLIC4 blocks SSc derived STAT1 activators. Serum depleted conditioned media was collected from primary healthy and SSc patient fibroblasts after 48 h. HaCaT were stimulated with the media for 48 h in the absence or presence of NPPB. pSTAT1, STAT1 pIRF3, pSMAD3 and CLIC4 protein levels were assessed by western blot. β-actin was used as a loading control. [file 13075_2025_3632_MOESM6_ESM.pdf]
