# Supplementary material for: Transforming growth factor beta (TGF-β) induces type 1 interferon signalling in systemic sclerosis keratinocytes through the chloride intracellular channel 4 (CLIC4)
Source: Arthritis Res Ther. 2025 Sep 1;27:173. doi: 10.1186/s13075-025-03632-6 (PMC12400655; doi:10.1186/s13075-025-03632-6)
Supplement: Supplementary file 2 — Supplementary Material 2. Supplementary Fig. 2: Knockdown of CLIC4 disrupts the pro-fibrotic phenotype in SSc dermal fibroblasts. Protein and RNA were extracted from healthy and SSc dermal fibroblasts transfected with siRNA specific for CLIC4. (A) pSMAD3, total SMAD3, β-catenin, GLI2, CTGF, α-SMA and CLIC4 protein levels were assessed by western blot. β-actin was used as a loading control. (B) Graph represent densitometry analysis for the mean and standard error for three independent experiments. CLIC4 (C), β-catenin (D) and GLI2 (E) transcript levels were assessed by qPCR. * p < 0.05, ** p < 0.01, *** p < 0.001. [file 13075_2025_3632_MOESM2_ESM.pdf]

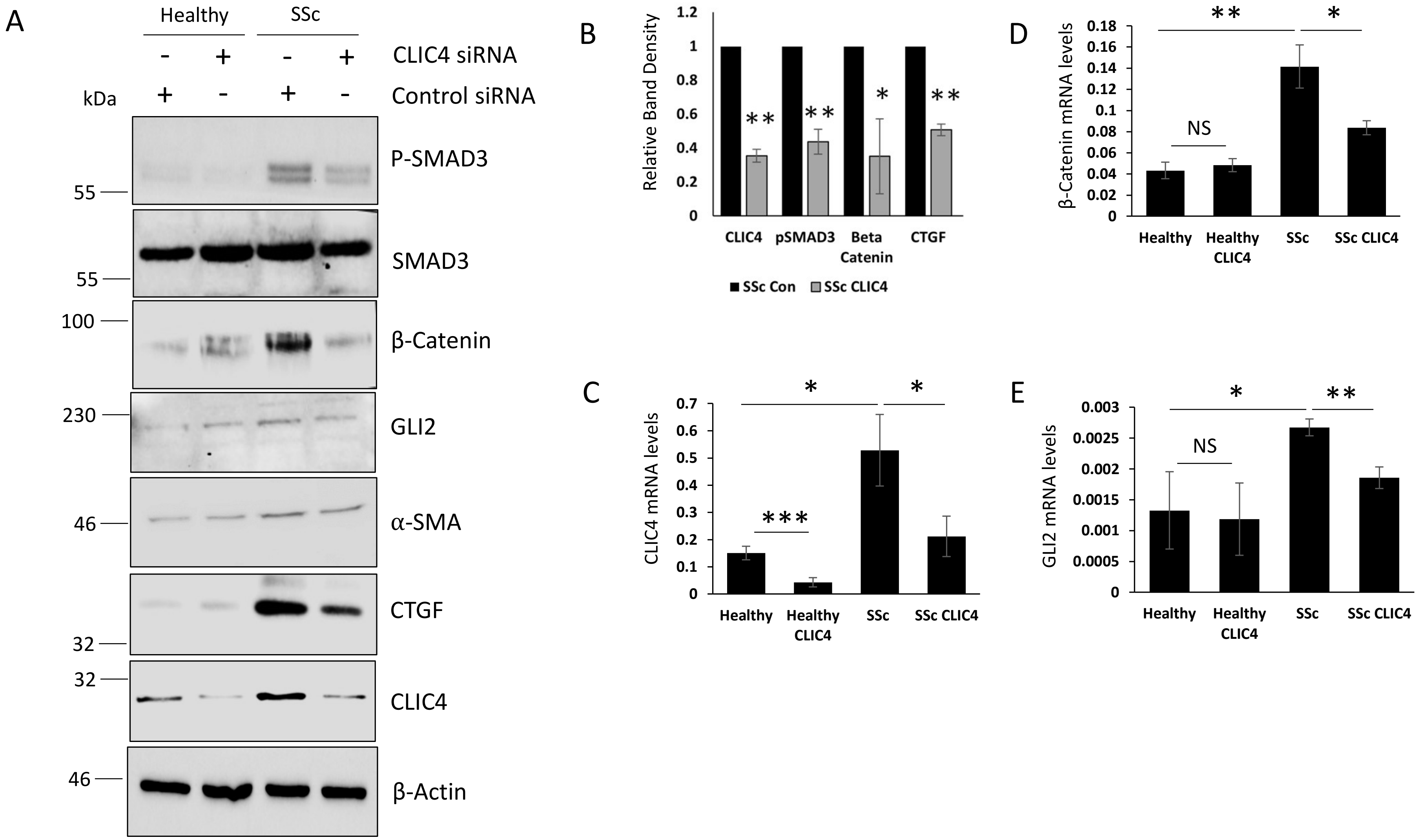

Supplementary Figure 2: Knockdown of CLIC4 disrupts the pro-fibrotic gene expression in SSc fibroblasts
