# Supplementary material for: Transforming growth factor beta (TGF-β) induces type 1 interferon signalling in systemic sclerosis keratinocytes through the chloride intracellular channel 4 (CLIC4)
Source: Arthritis Res Ther. 2025 Sep 1;27:173. doi: 10.1186/s13075-025-03632-6 (PMC12400655; doi:10.1186/s13075-025-03632-6)
Supplement: Supplementary file 4 — Supplementary Material 4. Supplementary Fig. 4: SSc fibroblast conditioned media induces IRF promoter activation in reporter cells. Thp1 dual luciferase reporter cells were grown in transwell co-culture with healthy and SSc dermal fibroblasts. Supernatant from the Thp1 cells was collected and IRF reporter activity was measured by QUANTI-Luc™ 4 Lucia/Gaussia. * p < 0.05, ** p < 0.01, *** p < 0.001. [file 13075_2025_3632_MOESM4_ESM.pdf]

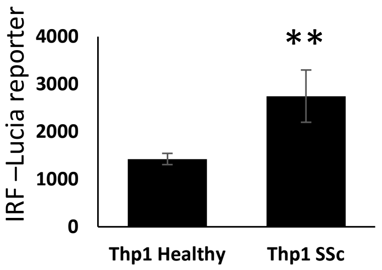

Supplementary Figure 4: SSc fibroblast conditioned media induces IRF promoter activation in reporter cells
