# Supplementary material for: Transforming growth factor beta (TGF-β) induces type 1 interferon signalling in systemic sclerosis keratinocytes through the chloride intracellular channel 4 (CLIC4)
Source: Arthritis Res Ther. 2025 Sep 1;27:173. doi: 10.1186/s13075-025-03632-6 (PMC12400655; doi:10.1186/s13075-025-03632-6)
Supplement: Supplementary file 5 — Supplementary Material 5. Supplementary Fig. 5: SSc fibroblast conditioned media induces CLIC4 nuclear expression in HaCaTs. HaCaT were stimulated with conditioned media from healthy and SSc dermal fibroblasts for 48 h. In addition HaCaTs were stimulated with TGF-β for 48 h. The cells were stained with an antibody specific for CLIC4 and visualized with an alexa-594 antibody. Nuclei were visualized with DAPI. [file 13075_2025_3632_MOESM5_ESM.pdf]

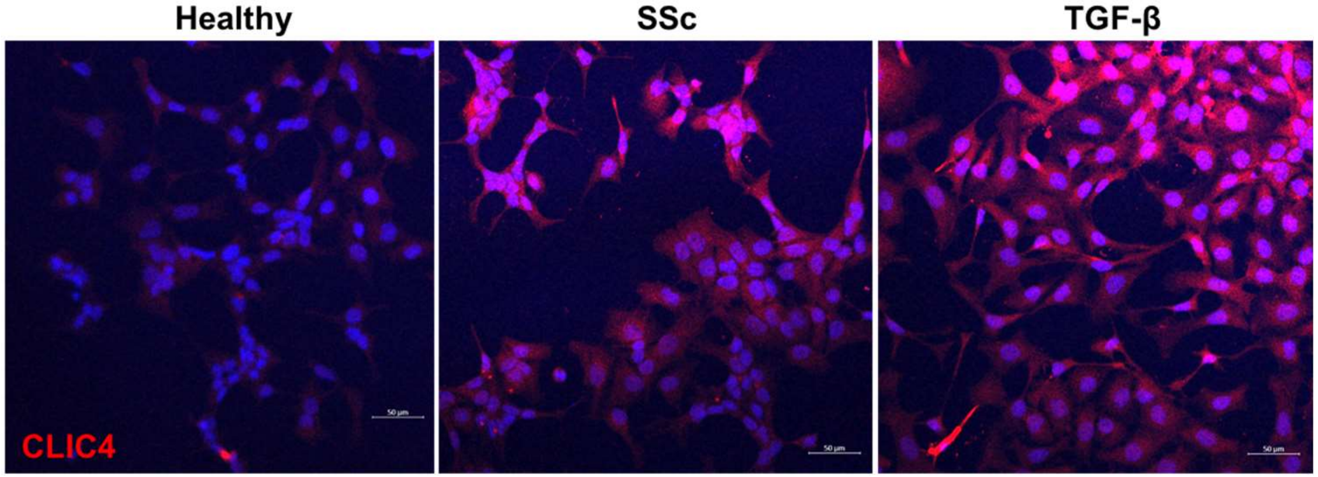

Supplementary Figure 5: SSc fibroblast conditioned media induces CLIC4 nuclear expression in HaCaTs
