# Supplementary material for: Transforming growth factor beta (TGF-β) induces type 1 interferon signalling in systemic sclerosis keratinocytes through the chloride intracellular channel 4 (CLIC4)
Source: Arthritis Res Ther. 2025 Sep 1;27:173. doi: 10.1186/s13075-025-03632-6 (PMC12400655; doi:10.1186/s13075-025-03632-6)
Supplement: Supplementary file 7 — Supplementary Material 7. Supplementary Fig. 7: SSc fibroblast exosomes do not stimulate CLIC4 expression in keratinocytes. Exosomes isolated from healthy and SSc patient fibroblasts were used to stimulate HaCaT for 48 h. (A) RNA was isolated from stimulated HaCaT and CLIC4 transcript levels were assessed. (B) Protein was isolated from stimulated HaCaT and CLIC4 protein levels were assessed by western blot. Graphs represent the mean and standard error for densitometry analysis. [file 13075_2025_3632_MOESM7_ESM.pdf]

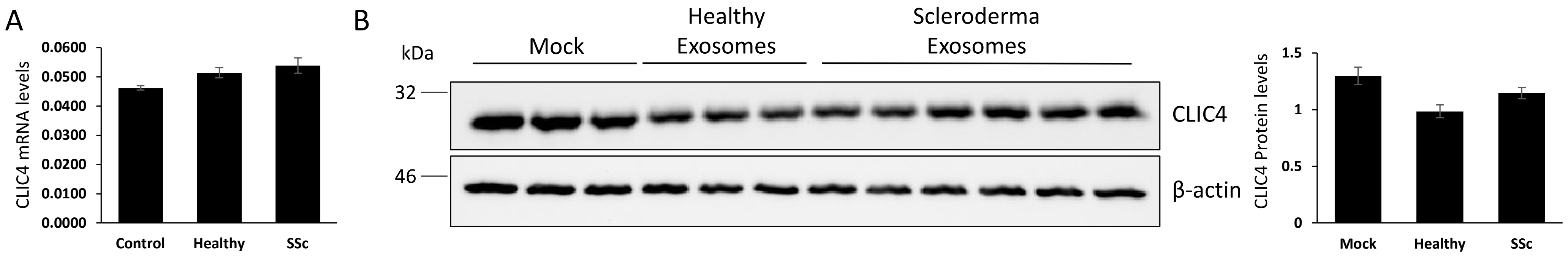

Supplementary Figure 7: SSc fibroblast exosomes do not stimulate CLIC4 expression in Keratinocytes
