# Supplementary material for: Transforming growth factor beta (TGF-β) induces type 1 interferon signalling in systemic sclerosis keratinocytes through the chloride intracellular channel 4 (CLIC4)
Source: Arthritis Res Ther. 2025 Sep 1;27:173. doi: 10.1186/s13075-025-03632-6 (PMC12400655; doi:10.1186/s13075-025-03632-6)
Supplement: Supplementary file 8 — Supplementary Material 8. Supplementary Fig. 8: Inhibition of the TGF-β signaling attenuates SSc fibroblast mediated activation of type 1 interferon in keratinocytes. Serum depleted conditioned media was collected from primary healthy and SSc patient fibroblasts after 48 h. HaCaTs were stimulated with the media for 48 h in the absence or presence of SD208. CXCL10 (A) and IFIT1 (B) transcript levels were assessed. * p < 0.05, ** p < 0.01, *** p < 0.001. [file 13075_2025_3632_MOESM8_ESM.pdf]

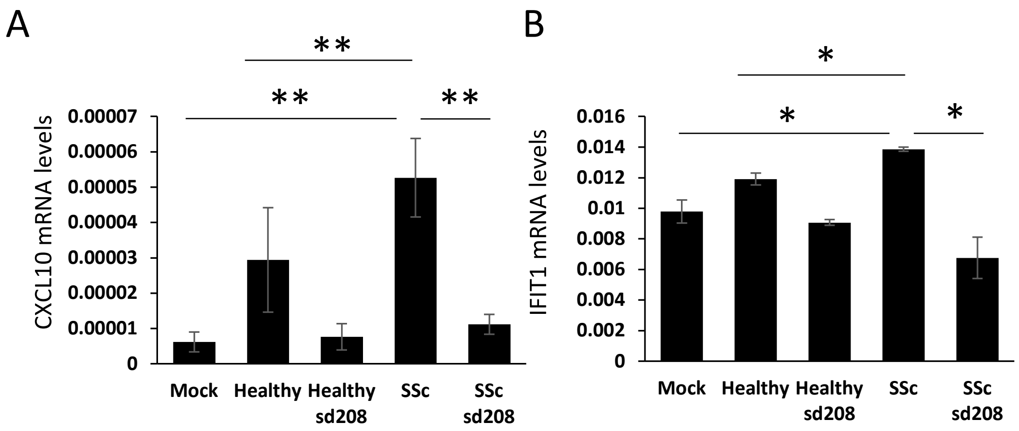

Supplementary Figure 8: Inhibition of TGF-β signalling attenuates SSc fibroblast mediated activation of type 1 interferon in keratinocytes
